# Reactive eccrine syringofibroadenoma triggered by thermal damage: case report^☆^^☆☆^

**DOI:** 10.1016/j.abd.2020.05.014

**Published:** 2021-01-25

**Authors:** Roberto Rheingantz da Cunha Filho, Lucas José Quioca, Graziela Junges Crescente Rastelli, José Fillus Neto

**Affiliations:** a School of Medicine, Universidade do Oeste de Santa Catarina, Joaçaba, SC, Brazil; b Universidade do Oeste de Santa Catarina, Joaçaba, SC, Brazil; c Department of Pathology, Faculdade Evangélica Mackenzie Paraná, Curitiba, PR, Brazil; d Department of Basic Pathology, Biological Sciences Sector, Universidade Federal do Paraná, Curitiba, PR, Brazil

Dear editor,

Eccrine syringofibroadenoma (ESFA) is a rare tumor that consists of the proliferation of ductal structures that resemble the acral portion of the eccrine sweat glands. It was first described by Mascaró in 1963, and there are just over 70 published cases in the literature.^1^, ^2^ Although histologically distinct, the clinical presentation of ESFA is variable, from a solitary papule, plaque, or nodule to multiple lesions, with a predilection for the limbs of older patients.^2^, ^3^ There is no consensus regarding its pathogenesis: tumor, hamartoma, or reactive hyperplasia.^4^ This report adds unprecedented aspects, as it demonstrates the association of ESFA with thermal damage and successful treatment using topical corticosteroids.

The authors report the case of a 71-year-old male, retired and without comorbidities. He reported the use of heat-generating plaster, consisting of iron powder, activated carbon, vermiculite, potassium chloride, and water (Fenaflan Patch®) to treat pain in the lateral region of the right thigh. However, there was a local reaction with burning and heat sensation, which caused the patient to remove the plaster before the indicated time. In the following days, he developed erythematous papules and a vegetating plaque, sometimes friable and bleeding, with mild pruritus. It progressed for three months, reaching the size of 4 cm (Fig. 1). A 5-mm punch biopsy was performed. The anatomopathological examination revealed anastomosing epithelial cords of cuboid cells forming trabeculae embedded in fibrous and myxoid stroma, which were highly vascularized from the epidermis to the deep dermis. The diagnosis of reactive type ESFA was confirmed (Fig. 2, Fig. 3).

**Fig. 1:**
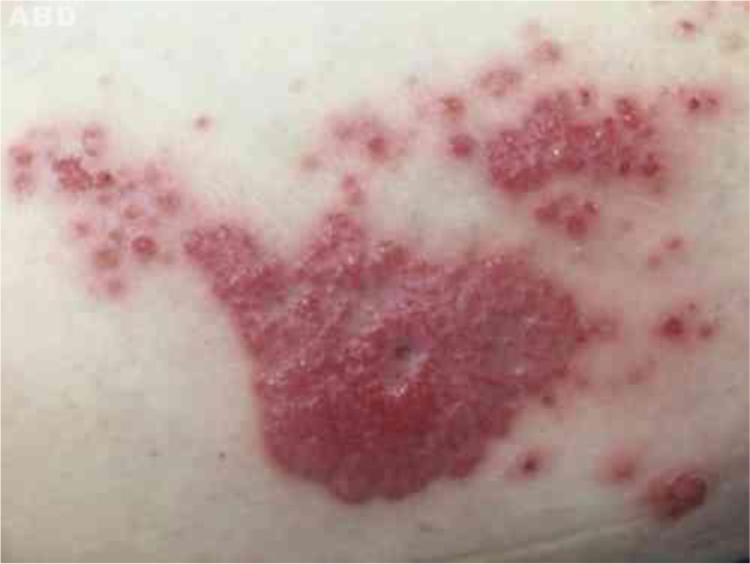
Vegetating plaque and erythematous and pinkish papules grouped in the lateral region of the right thigh.

**Fig. 2:**
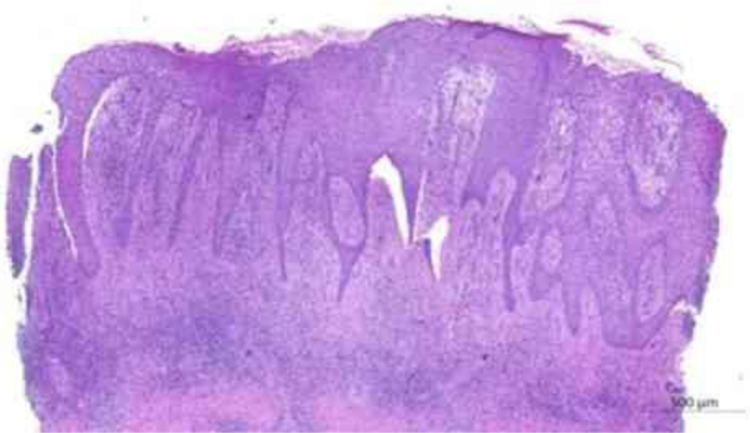
Anatomopathological examination: anastomosing epithelial cords of cuboid cells forming trabeculae in fibrous stroma that extend from the epidermis to the deep dermis (Hematoxylin & eosin, ×20).

**Fig. 3:**
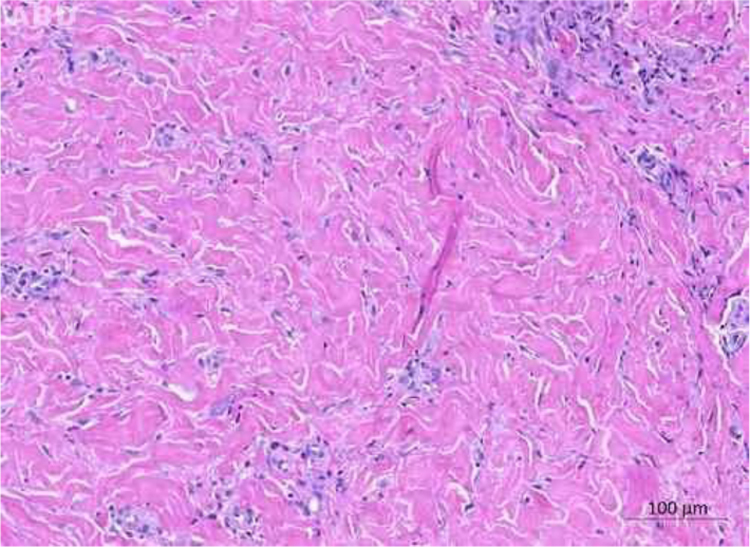
Anatomopathological examination: fibrous stroma in the middle and deep dermis (Hematoxylin & eosin, ×100).

Clobetasol in 0.05% cream was administered with occlusive application once a day for 20 days, with a fully effective response. The post-inflammatory residual erythema resolved completely within a few months.

The most current classification of ESFA includes five subtypes: solitary; multiple without skin changes; nevoid associated with ectodermal dysplasia; multiple associated with Schöpf syndrome; and the reactive subtype, associated with neoplastic or inflammatory dermatoses.^3^ The literature presents the occasional description with other entities, both inflammatory and neoplastic, including bullous pemphigoid, palmoplantar erosive lichen planus, psoriasis, congenital syphilis, nevus sebaceous, chronic skin ulcer, basal cell carcinoma, and squamous cell carcinoma.^2^

The reactive type is probably triggered by tissue damage and, in the process of repair and remodeling, there is hyperplasia in response to the damage.^3^ However, this is the first report in the literature to associate thermal injury as the triggering factor for ESFA.

Histopathology assessment is necessary for diagnostic confirmation, the eccrine ducts may or may not be demonstrated. Immunohistochemistry can help in difficult cases, with cells often positive for keratin 6 and 19, as well as filaggrin.^2^

There is no consensus regarding treatment. The literature presents reports of etretinate therapy, surgery, radiotherapy, and laser; however, there are no reports of treatment with clobetasol or other topical corticosteroids.^5^ The resolution of the present case occurred very quickly, with total regression in 20 days. The combination of the anti-inflammatory, antiproliferative, vasoconstrictor, and mitotic activity reduction effects of corticosteroids were probably determinant in the effective response to therapy.

## Financial support

None declared.

## Authors’ contributions

Roberto Rheingantz da Cunha Filho: Approval of the final version of the manuscript; design and planning of the study; drafting and editing of the manuscript; collection, analysis, and interpretation of data; effective participation in research orientation; intellectual participation in propaedeutic and/or therapeutic conduct of studied cases; critical review of the literature; critical review of the manuscript.

Lucas José Quioca: Approval of the final version of the manuscript; design and planning of the study; drafting and editing of the manuscript; collection, analysis, and interpretation of data; critical review of the literature; critical review of the manuscript.

Graziela Junges Ascending Rastelli: Approval of the final version of the manuscript; design and planning of the study; drafting and editing of the manuscript; collection, analysis, and interpretation of data; critical review of the literature; critical review of the manuscript.

José Fillus Neto: Approval of the final version of the manuscript; design and planning of the study; drafting and editing of the manuscript; collection, analysis, and interpretation of data; effective participation in research orientation; critical review of the literature; critical review of the manuscript.

## Conflicts of interest

None declared.
